# Gender, hyperandrogenism and vitamin D deficiency related functional and morphological alterations of rat cerebral arteries

**DOI:** 10.1371/journal.pone.0216951

**Published:** 2019-05-13

**Authors:** Éva Pál, Leila Hadjadj, Zoltán Fontányi, Anna Monori-Kiss, Norbert Lippai, Eszter M. Horváth, Attila Magyar, Eszter Horváth, Emil Monos, György L. Nádasy, Zoltán Benyó, Szabolcs Várbíró

**Affiliations:** 1 Institute of Clinical Experimental Research, Semmelweis University, Budapest, Hungary; 2 2nd Department of Obstetrics and Gynecology, Semmelweis University, Budapest, Hungary; 3 Department of Pathology, Jász-Nagykun-Szolnok County Hetényi Géza Hospital, Szolnok, Hungary; 4 Department of Physiology, Semmelweis University, Budapest, Hungary; 5 Department of Anatomy, Histology and Embryology, Semmelweis University, Budapest, Hungary; University of Mississippi Medical Center, UNITED STATES

## Abstract

Hyperandrogenism is a risk factor of cerebrovascular diseases as androgens can alter markedly the regulation of cerebrovascular tone. We examined the combined impact of androgen excess and vitamin D deficiency (VDD), a common co-morbidity in hyperandrogenic disorders, on remodeling and testosterone-induced vascular responses of anterior cerebral arteries (ACA) in order to evaluate the interplay between androgens and VDD in the cerebral vasculature. Male and female Wistar rats were either fed with vitamin D deficient or vitamin D supplemented diet. Half of the female animals from both groups received transdermal testosterone treatment. After 8 weeks, vessel lumen, wall thickness and testosterone-induced vascular tone of isolated ACA were determined using pressure microangiometry and histological examination. Androgen receptor protein expression in the wall of cerebral arteries was examined using immunohistochemistry. In female rats only combined VDD and testosterone treatment decreased the lumen and increased the wall thickness of ACA. In males, however VDD by itself was able to decrease the lumen and increase the wall thickness. Vascular reactivity showed similar alterations: in females, testosterone constricted the ACA only after combined VDD and hyperandrogenism, whereas in males VDD resulted in increased testosterone-induced contractions in spite of decreased androgen receptor expression. In conclusion, a marked interplay between hyperandrogenism and VDD results in inward remodeling and enhanced testosterone-induced constrictions of cerebral arteries, which might compromise the cerebral circulation and thus, increase the risk of stroke in the long term. In addition, the early cerebrovascular manifestation of VDD appears to require androgen excess and thus, depends on gender.

## Introduction

Sex steroids have considerable impact on the cerebral circulation [1] since endogenous and exogenously administered gonadal hormones influence the cerebrovascular tone and blood perfusion under physiological and pathophysiological conditions [2]. The impact of estradiol on the cerebral circulation is well known, however, the effect of testosterone on cerebral vessels is more obscure [1, 2]. Nevertheless, testosterone could be responsible—at least partly—for the increased risk of cerebrovascular diseases in men [1, 2] and in hyperandrogenic women as compared to premenopausal healthy women [3].

Effectiveness of vitamin D (VitD) supplementation in the prevention of cardiovascular events is obscure [4], however, vitamin D deficiency (VDD) appears to be linked to the metabolic syndrome as well as to cardiovascular diseases including hypertension, atherosclerosis and cerebrovascular disturbances in both genders [5, 6]. VDD can impair vessel morphology and reactivity [7, 8], probably due to both alterations of gene expression and non-genomic actions [5]. The role of VitD in the modulation of arterial function has already been described in several vessel types [8–10]; for instance, both animal [7] and human studies [11] imply the presence of cerebrovascular impairment in VDD.

Interestingly, 67–85% of women with androgen excess, particularly with polycystic ovary syndrome (PCOS), are affected by VDD [12], which appears to influence the development of PCOS due to the alterations of gene transcription and hormonal regulation [12, 13]. In addition, several studies report that VDD can worsen the cardiovascular manifestation of PCOS [12]. Both VDD and hyperandrogenism appear to be associated with cerebrovascular disorders [14–16] partly due to impaired vascular functions [10, 17]. Therefore, a significant interplay is assumable between VitD and androgens in the cerebral circulation, which might also explain the gender differences in the vascular manifestation of VDD [8].

In the present study, we hypothesized an interplay between VDD and hyperandrogenism, assuming a significant role for the vascular effect of androgens in this interaction, which could lead to early alterations in cerebrovascular morphology and reactivity predicting the manifestation of stroke in the long term. Thus, we aimed to analyze the impact of testosterone (endogenous and exogenously administered) and VDD on the morphological and functional properties of anterior cerebral arteries (ACA) in a rodent model.

## Materials and methods

### Experimental animal model

Four-week-old Wistar rats (22 males and 46 females) were involved in the experiments. Animals were housed at constant temperature (22 ± 1°C) and 12–12 hours light-dark cycle. 11 male and 22 female rats were sorted out randomly and fed with VDD diet (EF R/M, E15312-24, ssniff Spezialdiäten GmbH, Soest, Germany) for eight weeks (*♂d*- and *♀d*- groups, respectively). All other animals received conventional rat chow (SM R/M, S8106-S011, sniff Spezialdiäten GmbH, Soest, Germany) with per os VitD supplementation (Vigantol, 20.000 IU/mL cholecalciferol (Merck Serono, Mumbai, India)) providing optimal VitD supply: the daily VitD intake was 300 IU/100 g b. w. which provided 26.31±2.21 ng/mL serum 25-hydroxyvitamin D level, whereas VDD diet resulted in 5.63±0.46 ng/mL 25-hydroxyvitamin D level after eight week of treatment. Half of the female rats from both the VitD sufficient and deficient groups received transdermal testosterone treatment for eight weeks (*T♀D+* and *T*♀*D-* groups, 0.033 mg/g b. w. testosterone (Androgel 1%, Laboratories Besins International S.A., Paris, France)), which increased serum total testosterone levels from 0.46±0.11 ng/mL to 3.61±0.37 ng/mL (the average testosterone level of male rats was 5.87±0.60 ng/mL).

Table 1 represents the experimental design for all groups. Body weight was measured regularly, and the gain of body weight was calculated. At the age of twelve weeks, blood pressure of the animals was measured by cannulation of the carotid artery under general anesthesia (pentobarbital, 45 mg/kg b.w., i. p.; Ceva-Phylaxia, Budapest, Hungary). After perfusion *via* the carotid artery with heparinized Krebs-Ringer solution and decapitation under anesthesia, the brain was removed, and ACA segments were prepared under a stereomicroscope (Wild M3Z, Heerbrugg, Switzerland).

**Table 1:** Experimental design.

SEX	♀				♂	
VITAMIN D	+		-		+	-
TESTOSTERONE TREATMENT	+	-	+	-	-	-
**SYMBOL**	*T♀D+* (n = 12)	*♀D+* (n = 12)	*T ♀d-* (n = 11)	♀*D-* (n = 11)	♂*D+* (n = 11)	♂*D-* (n = 11)

♀*D+* and ♀*D-* stand for female rats that received conventional rat chow and vitamin D deficient diet, respectively. *T*♀*D+* and *T*♀*d-* symbolize testosterone treated females with vitamin D supply and with vitamin D deprivation, respectively. ♂*D+* and ♂*D-* stand for male rats that received conventional rat chow and vitamin D deficient diet, respectively.

All procedures conformed to the Guide for the Care and Use of Laboratory Animals published by the US National Institutes of Health (8th edition, 2011) and the EU-conform Hungarian Law on Animal Care (XXVIII/1998). The Institutional Animal Care and Use Committee of Semmelweis University approved the study protocol (PEI/001/820-2/2015). All surgery was performed under sodium pentobarbital anesthesia, and all efforts were made to minimize suffering.

### Pressure microangiometry

An approximately 2-mm-long segment of the ACA was isolated and excised, and its morphological and functional properties were examined using pressure microangiometry [7]. After equilibration at 50 mmHg intraluminal pressure in normal Krebs-Ringer solution [7], cumulatively increasing concentrations of testosterone (Sigma-Aldrich, Darmstadt, Germany) were applied (10^−9^ mol/L—10^−6^ mol/L, 5 min incubation for each step), considering the appropriate plasma concentrations of the hormone in males and females [10, 18]. Pictures were taken during the experiment by a digital camera (Leica DFC 320) connected to an inverted microscope (Leica, Wetzlar, Germany). The outer and inner diameters of the vessels were measured using an ImageJ image analyzing software (Image J 1.5 NIH, USA). For calibration, a micrometer etalon (Wild, Heerbrugg, Switzerland) was applied. Testosterone-induced tone was expressed as 100*(Ri_*nKR*_*-Ri*_*TEST*_*)/Ri*_*nKR*,_ where *Ri*_*TEST*_ is the inner radius after incubation with testosterone and *Ri*_*nKR*_ is the inner radius in normal Krebs-Ringer solution.

### Histology and immunohistochemistry

Ovaries and ACA segments were fixed freshly with formalin for histological examination; thereafter, ovaries were stained with hematoxylin and eosin, whereas ACA segments were either stained also with hematoxylin and eosin or immunostained for androgen receptor (AR). For immunostaining, artery segments were incubated at 37°C for 36 min with polyclonal rabbit ChIP Grade anti-androgen receptor antibody (ab74272, AB_1280747; Abcam, Cambridge, MA, USA) [19] using the Ventana Benchmark Ultra System after deparaffinization and antigen retrieval (97°C, 8 min). The UltraView Universal DAB Detection Kit (Ventana Medical Systems, Inc., Tucson, AZ, USA) was used for detecting primary antibodies. Data collections were made by a microscope (Zeiss AxioImager.A1) coupled with a video-camera (Zeiss AxioCAm MRc5 CCD), and pictures were analyzed with ImageJ image analyzing software (Image J 1.5 NIH, USA). The wall thickness of arteries was determined on hematoxylin and eosin stained segments. Pictures of AR staining were analyzed using the „Color deconvolution” profile of ImageJ. The percentage of positively stained tissue area to total area of the section (area %) was calculated.

### Statistical analysis

All data are presented as mean ± SEM. Normal distribution of datasets was checked with Shapiro-Wilk test. Statistical analysis for the artery geometry of males was performed using Student’s t test, whereas for any other parameters two-way ANOVA followed by Tukey’s post hoc test was used, and p<0.05 was considered statistically significant. GraphPad Prism version 6.0 was used for statistical analysis.

## Results

### Gain of body weight, blood pressure and ovarian histology

**Fig 1:**
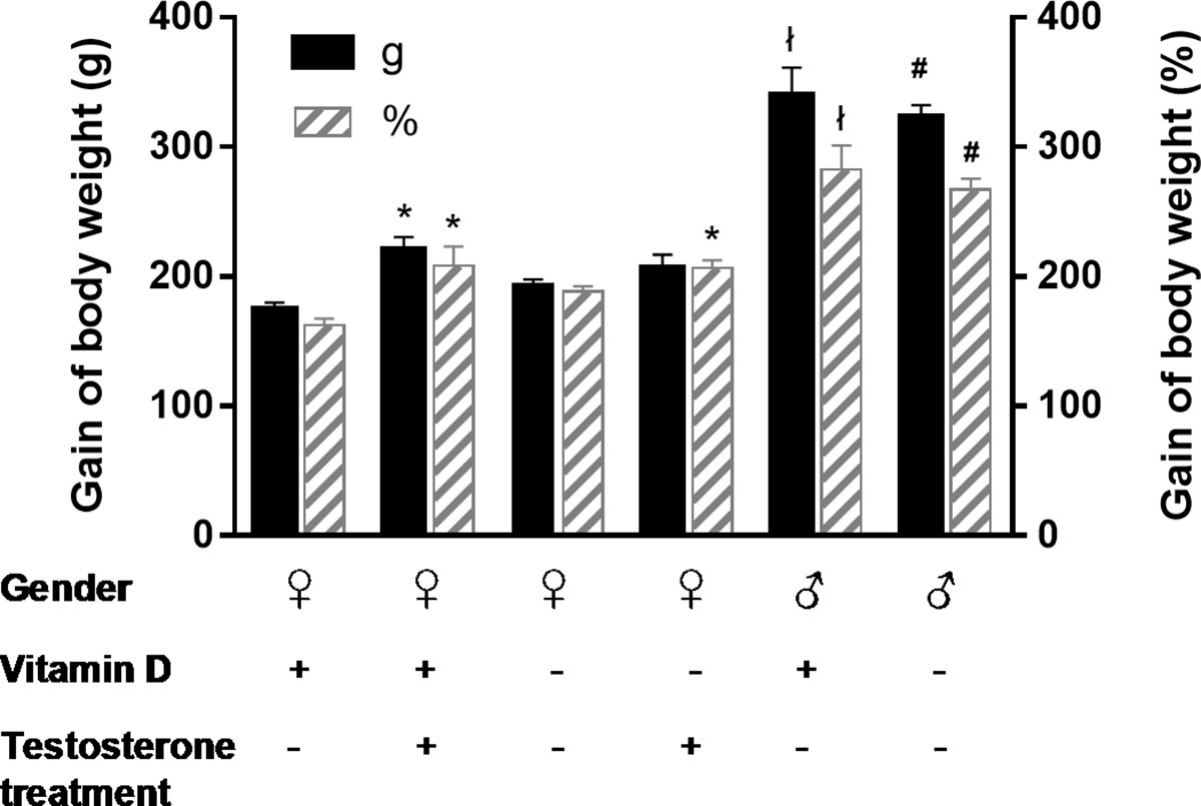
Gain of body weight. Hyperandrogenism enhanced the weight gain of female rats (*p = 0.0031, 0.0047 and 0.0067 vs. *♀D+* for *T♀D+* (g), *T♀D+* (%*)* and *T♀D-* (%), respectively; n = 10–11; two-way ANOVA followed by Tukey’s post hoc test). Male rats had increased weight gain as compared to females (^**ł**^p<0.0001 vs. *♀D+*, #p<0.0001 vs. *♀D-*; n = 10–11; two-way ANOVA followed by Tukey’s post hoc test). VDD influenced the weight gain neither in female nor in male animals. Gain of body weight (g) = final body weight (g)—initial body weight (g). Gain of body weight (%) = 100*(final body weight (g)—initial body weight (g))/initial body weight (g).

**Fig 2:**
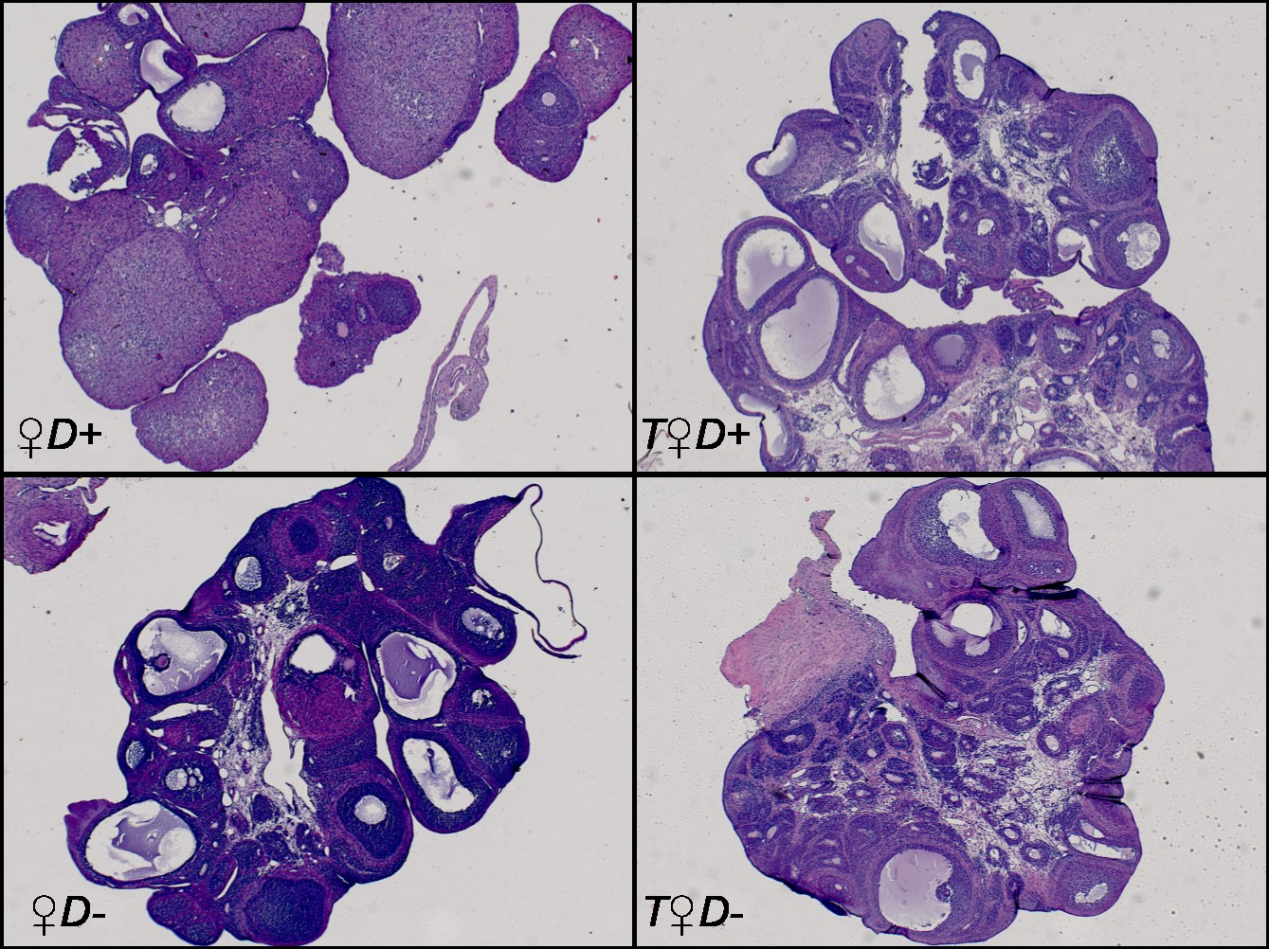
Ovarian histology. Representative images of ovaries stained with hematoxylin and eosin. The ovaries of *T♀D+* and *T♀D*- animals are characterized by increased number of small-sized primordial follicles resembling the manifestation of PCOS.

Male and hyperandrogenic female rats had enhanced weight gain as compared to females (Fig 1), indicating that the gain of body weight was impacted by gender and hyperandrogenism. Vitamin D status, however, did not influence weight gain (Fig 1). Arterial blood pressure was not affected by either treatment: mean arterial pressure was 130.5±2.7 mmHg in male whereas 116.2±6.3 mmHg in female and 111.1±2.0 mmHg in hyperandrogenic female rats. Vitamin D status had no influence on blood pressure as well. The ovaries were stained with hematoxylin and eosin for histological examination. Hyperandrogenic female rats had multiple small-sized primordial follicles in the ovaries (Fig 2), indicating that hyperandrogenism impairs follicle maturation independently from vitamin D status.

### Arterial geometry

**Fig 3:**
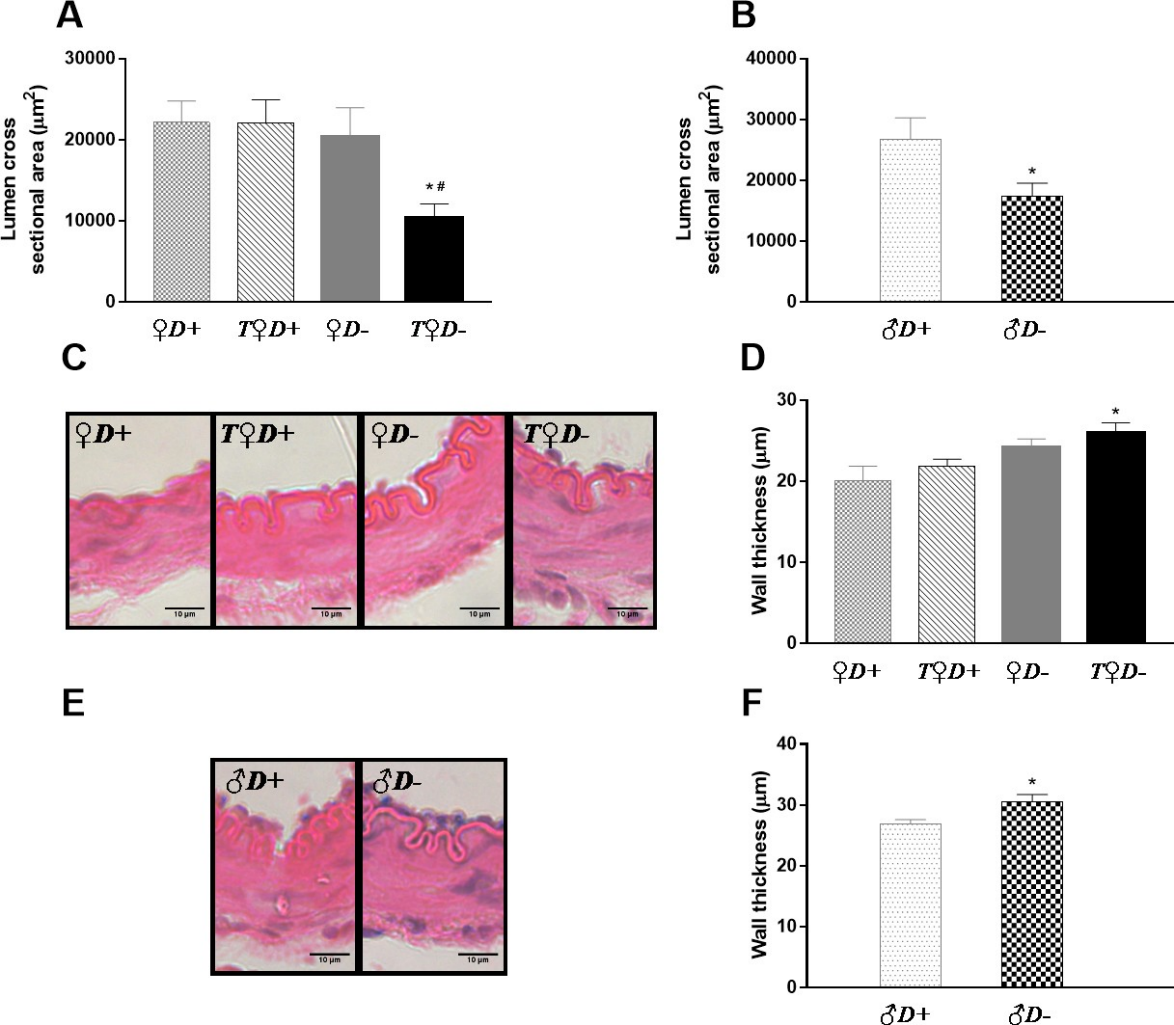
Cerebral artery geometry. (A) The combined effect of VDD and hyperandrogenism significantly decreased the lumen cross sectional area of female rats (*p = 0.027 vs. *♀D+;* ^#^p = 0.034 vs. *T♀D+*; n = 10–11; two-way ANOVA followed by Tukey’s post hoc test). (B) In males, VDD resulted in decreased lumen cross sectional area under physiological conditions (*p = 0.033 vs. *♂D+*, n = 10–11; Student’s t test). (C) Representative images of cerebral arteries of female rats stained with hematoxylin and eosin. (D) The combined effect of VDD and androgen excess caused an increase in wall thickness of female rats (*p = 0.010 vs. *♀D+;* n = 5–7; two-way ANOVA followed by Tukey’s post hoc test). (E) Representative images of cerebral arteries of male rats stained with hematoxylin and eosin. (F) VDD significantly increased the wall thickness of arteries in male rats (*p = 0.032 vs. *♂D+*, n = 5–7; Student’s t test).

Morphological parameters of the cerebral arteries were measured under physiological conditions. No statistically significant difference was found in the lumen cross sectional area as a function of gender (22173±2602 μm^2^ and 26745±3542 μm^2^ for *♀D+* and *♂D+* groups, respectively). In female rats neither VDD nor androgen excess resulted in a significant change of the lumen cross sectional area (Fig 3A). Surprisingly, however, combined VDD and hyperandrogenism decreased markedly the lumen of cerebral arteries of female rats (Fig 3A), indicating that VDD results in increased active tension and/or inward remodeling only in the presence of testosterone. Accordingly, ACAs of *♂D-* rats had significantly decreased lumen cross sectional area as compared to those of *♂D+* animals (Fig 3B).

To gain further insight into the mechanism (increased vascular tone vs. remodeling) responsible for the above-mentioned alterations in vessel lumen, the wall thickness of arteries was determined on hematoxylin and eosin stained sections of the ACA (Fig 3C and 3E). VDD caused an increase in the wall thickness of males (Fig 3F); however, in females, wall thickness was only increased by combined VDD and androgen excess (Fig 3D). These results indicate that the increase in wall thickness could be responsible for the observed changes in cross sectional area. In addition, this VDD-induced inward remodeling appears to require the presence of androgens.

### Vasoactive effects of acute testosterone application

**Fig 4:**
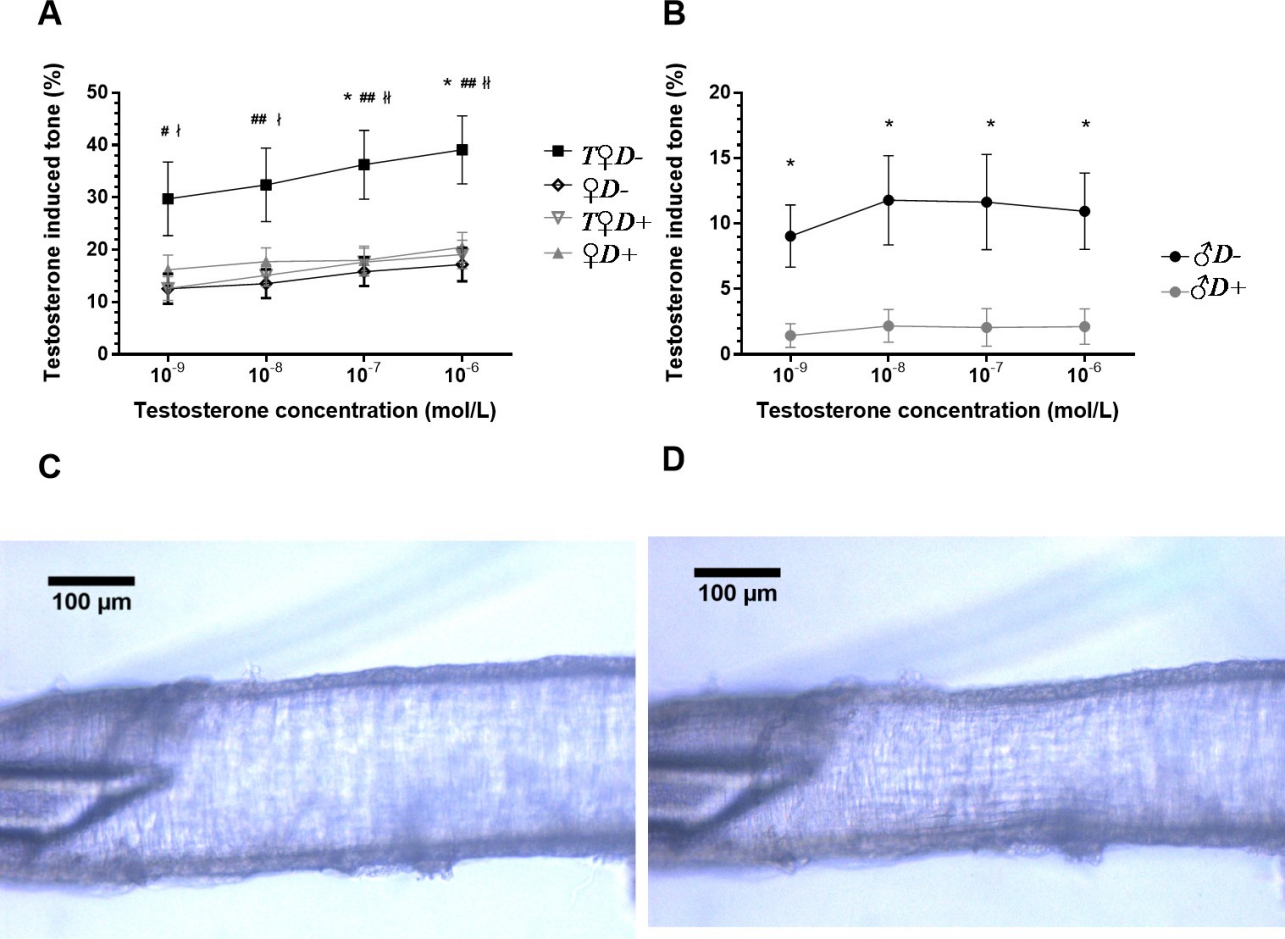
Testosterone-induced acute vascular responses. (A) Acute testosterone application resulted in increased vasoconstriction in the ACA of female rats subjected to combined VDD and hyperandrogenism (*T♀D-*) (*p = 0.015 and 0.016 vs. *♀D+* for 10^−7^ and 10^−6^ mol/L concentration, respectively; **#**p = 0.024 vs. *♀D-*, **##**p = 0.009, 0.004 and 0.003 vs. *♀D-* for 10^−8^, 10^−7^ and 10^−6^ mol/L concentration, respectively; ^ł^p = 0.017 and 0.013 vs. *T♀D+* for 10^−9^ and 10^−8^ mol/L concentration, respectively; ^łł^p = 0.007 and 0.004 vs. *T♀D+* for 10^−7^ and 10^−6^ mol/L concentration, respectively; n = 10–11; two-way ANOVA followed by Tukey’s post hoc test). (B) In males, VDD significantly increased the testosterone-induced tone (*p = 0.030, 0.012, 0.012 and 0.025 vs. *♂D+* for 10^−9^, 10^−8^, 10^−7^ and 10^−6^ mol/L concentration, respectively; n = 10–11; two-way ANOVA followed by Bonferroni’s post hoc test). (C-D) Representative images of the anterior cerebral artery showing the testosterone induced vasoconstriction in female rats subjected to combined VDD and androgen excess. (C) Anterior cerebral artery segment under physiological conditions (in normal Krebs-Ringer solution). (D) Anterior cerebral artery segment after the application of 10^−6^ mol/L testosterone. Testosterone-induced tone (%) = 100*(Ri_*nKR*_*-Ri*_*TEST*_*)/Ri*_*nKR*,_ where *Ri*_*TEST*_ is the inner radius after incubation with testosterone and *Ri*_*nKR*_ is the inner radius in normal Krebs-Ringer solution.

Testosterone has been reported recently to evoke acute vasoactive effects, although with high variability depending on species and vascular region [20–23]. In our present study, testosterone appeared to induce stronger constrictions of the ACA in females as compared to males. Interestingly, in vessels of *T♀D+* and *♀D-* animals the contractile effect of testosterone remained unaltered as compared to *♀D+* rats (Fig 4A). In contrast, combined VDD and androgen excess resulted in a more than two-fold increase of testosterone-induced vasoconstriction (Fig 4A). In accordance, testosterone caused vasoconstriction of arteries prepared from *♂D-* but not from *♂D+* rats (Fig 4B). Therefore, the acute vasoactive effect of testosterone appears to depend both on gender and VitD status, and hyperandrogenic VitD-deficient female animals are the most prone to cerebrovascular constriction.

### Androgen receptor immunohistochemistry

**Fig 5:**
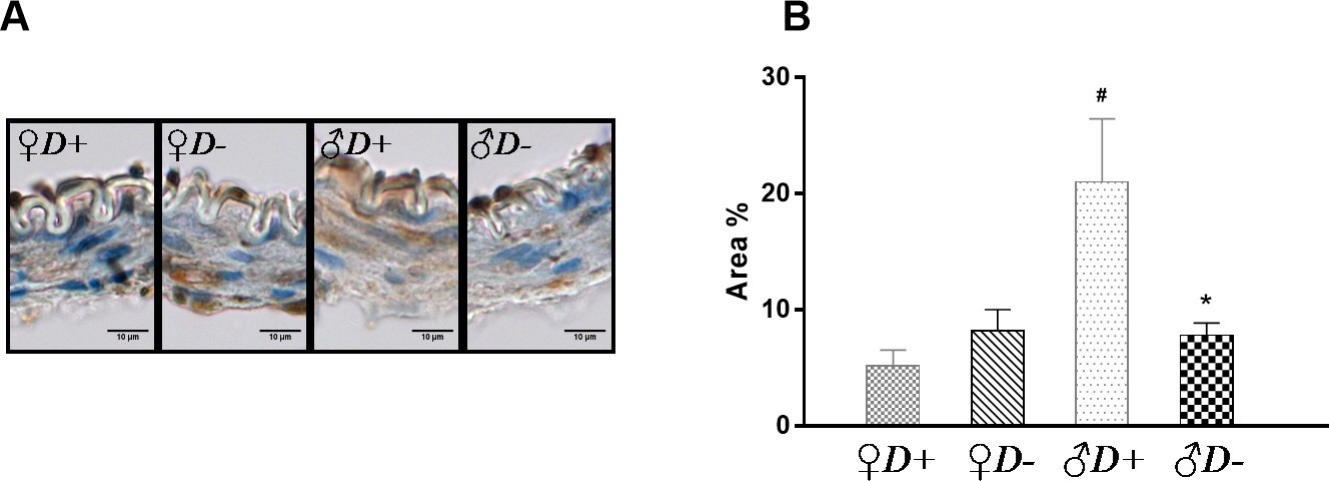
Evaluation of immunohistochemical staining of arteries. (A) Representative immunohistochemical images of cerebral arteries stained for AR. (B) In females, VDD did not alter the expression of the AR protein in the vessel wall of arteries; in contrast, in males VDD significantly decreased the expression of AR (*p = 0.008 vs. *♂D+*, n = 4–7; two-way ANOVA followed by Tukey’s post hoc test). In addition, the expression of the AR protein was impacted by gender: expression significantly decreased in females as compared to males (**#**p = 0.016 vs. *♀D+;* n = 4–4; two-way ANOVA followed by Tukey’s post hoc test).

Finally, to examine the possible role of VitD in the modulation of cerebrovascular AR protein expression, the percentage of positively stained area in the vessel wall was determined. The expression of AR protein was not affected by VDD in females, however it was higher in the vessel wall of VitD-sufficient males as compared to females (Fig 5), implying a marked gender difference in the protein expression level of AR in cerebral arteries. Interestingly, a significant decrease in AR protein expression was determined in *♂D-* as compared to *♂D+* animals (Fig 5).

## Discussion

The association between vascular disorders and VDD [5, 6, 24, 25] as well as androgen excess [3, 26] has long been known, however the combined effect of those on cerebral arteries and the mechanism responsible for the increased risk of stroke has not been completely revealed yet. The results we present here indicate for the first time a marked interplay between androgens and VDD in the cerebral circulation characterized by an increased vascular reactivity to androgens. Interestingly, cerebrovascular remodeling induced by VDD appears to require high androgen levels, which may explain the relative resistance of VitD-sufficient females to cerebrovascular disorders as compared to males. In addition, combined VDD and hyperandrogenism result in increased testosterone-induced vasoconstriction, which may compromise the maintenance of optimal cerebrovascular tone under physiological conditions [2].

Hyperandrogenism may lead to cerebrovascular impairment [27] probably due to the long-term genomic effects of excess androgen [2]. VDD is also linked to cerebrovascular disorders particularly *via* impaired vessel morphology and reactivity [2, 6–8, 28–30]. In our study, however, neither VDD nor androgen excess alone caused alterations in vessel lumen and wall thickness of female rats, indicating that neither disorder alone causes remodeling in the cerebral arteries of females, at least not within 8 weeks. On the contrary, some studies report that androgen excess leads to the alteration of either diameter or wall thickness in peripheral vessels [31–33], therefore the impact of hyperandrogenism on vascular remodeling may depend on vessel type. Surprisingly, in males the endogenous physiological androgen level together with VDD decreased the lumen and increased the wall thickness of cerebral arteries. Similar remodeling was observed in females with combined VDD and androgen excess. These results indicate that androgen excess must be necessary for the development of the cerebrovascular consequences of VDD, implying a possible interaction between these two factors. VDD appears to induce similar alterations in hyperandrogenic females to those in males, however the mechanism might depend on gender. Cerebrovascular contractility of arteries of VDD males was shown to increase [7], but in hyperandrogenic females VDD had no such effect [34]. Therefore, in males, in addition to arterial remodeling, the enhanced vascular reactivity to vasoconstrictor agents could contribute to the decreased cerebral vessel lumen indicating that gender differences might alter the cerebrovascular manifestation of VDD. VDD and hyperandrogenism have been reported to be associated with hypertension [6, 25, 35], which could induce cerebrovascular alterations, however, neither treatment impacted the blood pressure of animals in our present study. Therefore, we could exclude the possibility that the observed vascular changes would be secondary consequences of VDD/hyperandrogenism-induced hypertension.

Several studies report an interplay between androgens and VitD in the regulation of prostate cancer cells [36–38], chondrocyte [39] and vascular cell proliferation [40]. The interaction might be attributed to a cross-talk between AR- and VitD-receptor-mediated gene expression, as VitD and androgens have many common target genes, the transcription of which they modulate together [41, 42]. Furthermore, several transcription factor-mediated signaling pathways are only active, when both hormones are present [42], and VitD also appears to impact the expression of AR [36, 39]. Therefore, we determined the expression of the AR protein in the cerebral arteries of VDD animals. VDD resulted in decreased AR protein expression in the artery wall of males but, surprisingly, not in that of females. The decreased protein expression might be deleterious in males, as endogenous androgens may protect against vascular remodeling [43, 44], atherogenesis [44–46] and thrombosis [47]; furthermore, it could also contribute to the preservation of NO bioavailability [43]. On the contrary, in females, VDD did not alter AR protein expression, which supports the findings that (i) short-term VDD alone does not cause alterations in cerebral vessels of females, and (ii) men are more seriously affected by the cerebrovascular consequences of VDD than healthy premenopausal women.

Long-term treatment with androgens results in increased vascular tone in females [48] as well as in male orchiectomized rats [49]. However, testosterone is reported to have also rapid short-term vasoactive effects [2], particularly in peripheral vessels [21–23]. *Ex vivo*, direct application of pharmacological doses of testosterone appears to evoke a rapid decrease in vascular tone [1]–due to the activation of an uncharacterized “non-classical” membrane-bound receptor or to an interaction with ion channels [2]. The impact of acute testosterone application on cerebral arteries and particularly the effect of chronic androgen excess and VDD on the rapid short-term actions of testosterone, however, have not been revealed yet. As androgens contribute markedly to the cerebrovascular manifestation of VDD, their acute vascular effect might also be altered by VDD. Therefore, we analyzed the vascular effect of acute testosterone application on cerebral arteries and its dependence on VitD and androgen status. In female animals, similarly to the alterations in vessel morphology, only the combined effect of VDD and androgen excess enhanced the testosterone-induced vascular tone, independently from the applied concentrations. However, in VDD males testosterone caused marked contractions, which could probably be a consequence of the increased vascular contractility in VDD [7]. These results imply that androgen excess in females or physiological androgen levels in males are a prerequisite for the enhancement of acute testosterone-induced tone in VDD. In addition, the effect of testosterone on cerebral arteries might depend on gender under normal VitD and physiological androgen status: acute testosterone application caused cerebral vasoconstriction in females, but not in males. Thus, physiological concentrations of testosterone (in the nanomolar range) [18] do not compromise the cerebral circulation in males. A similar neutral effect was determined in coronary arteries: testosterone did not change the vessel tone [22] [23], only impaired endothelium-dependent relaxation [22] and potentiated agonist-induced contraction [23]. On the contrary, in the present study both physiological (10^−9^ mol/L) and supraphysiological concentrations (10^−8^–10^−6^ mol/L) of testosterone [18] induced vasoconstrictions in females. These results indicate that, in addition to vascular remodeling, the testosterone-induced tone caused by VDD and hyperandrogenism might lead to cerebrovascular disorders, as sex steroids may contribute markedly to the maintenance of cerebrovascular tone and reactivity [2].

Both hyperandrogenism and VDD are associated reportedly with cerebrovascular disorders [14, 15]; however, the present study shows that they synergistically cause deleterious alterations in the morphology and reactivity of ACA within a relatively short time, which could facilitate the development of cerebrovascular diseases or aggravate their outcomes. Although cerebral infarctions localized in the frontal territory are relatively rare, impaired regulation of the ACA could lead to serious cognitive and executive impairments [50], which might be aggravated by combined VDD and androgen excess. However, in addition to ACA, both disorders impair presumably other vascular regions of cerebral circulation including the microcirculation, which might contribute for instance to blood-brain barrier disruption after stroke as it was reported in VDD by a recent study [51]. Surprisingly, elevated androgen levels appear to be required for the early cerebrovascular manifestation of VDD, which might imply that post-menopausal and hyperandrogenic women are at increased risk of the development of stroke induced by VDD. These findings underline the importance of the prevention of VDD in humans and encourages further examination of the interplay between VDD and androgen excess in cardiovascular disorders.

## Conclusions

The present study demonstrates a marked interplay between androgen excess and VDD in the cerebral circulation, which impairs the morphology and reactivity of cerebral arteries and may therefore lead to stroke in the long term. We propose that the cerebrovascular manifestation of VDD requires androgens and is thus gender-dependent. In addition, the combined effect of VDD and hyperandrogenism appears to lead to impaired testosterone-induced vascular responses, which might compromise the cerebral circulation.

## References

[1] Krause DN, Duckles SP, Gonzales RJ. Local oestrogenic/androgenic balance in the cerebral vasculature. Acta Physiol (Oxf). 2011;203(1):181–6. 10.1111/j.1748-1716.2011.02323.x PMC3156882 21535417

[2] Gonzales RJ. Androgens and the cerebrovasculature: modulation of vascular function during normal and pathophysiological conditions. Pflugers Arch. 2013;465(5):627–42. 10.1007/s00424-013-1267-3 23605065

[3] Fauser BC, Tarlatzis BC, Rebar RW, Legro RS, Balen AH, Lobo R, et al Consensus on women's health aspects of polycystic ovary syndrome (PCOS): the Amsterdam ESHRE/ASRM-Sponsored 3rd PCOS Consensus Workshop Group. Fertil Steril. 2012;97(1):28–38.e25. 10.1016/j.fertnstert.2011.09.024 22153789

[4] Manson JE, Cook NR, Lee IM, Christen W, Bassuk SS, Mora S, et al Vitamin D Supplements and Prevention of Cancer and Cardiovascular Disease. N Engl J Med. 2019;380(1):33–44. 10.1056/NEJMoa1809944 30415629 PMC6425757

[5] Holick MF. Vitamin D deficiency. N Engl J Med. 2007;357(3):266–81. 10.1056/NEJMra070553 17634462

[6] Norman PE, Powell JT. Vitamin D and cardiovascular disease. Circ Res. 2014;114(2):379–93. 10.1161/CIRCRESAHA.113.301241 24436433

[7] Pal E, Hadjadj L, Fontanyi Z, Monori-Kiss A, Mezei Z, Lippai N, et al Vitamin D deficiency causes inward hypertrophic remodeling and alters vascular reactivity of rat cerebral arterioles. PLoS One. 2018;13(2):e0192480 10.1371/journal.pone.0192480 29408903 PMC5800593

[8] Tare M, Emmett SJ, Coleman HA, Skordilis C, Eyles DW, Morley R, et al Vitamin D insufficiency is associated with impaired vascular endothelial and smooth muscle function and hypertension in young rats. J Physiol. 2011;589(Pt 19):4777–86. 10.1113/jphysiol.2011.214726 21807617 PMC3213423

[9] Sara L, Nadasy GL, Antal P, Monori-Kiss A, Szekeres M, Masszi G, et al Pharmacological reactivity of resistance vessels in a rat PCOS model—vascular effects of parallel vitamin D(3) treatment. Gynecol Endocrinol. 2012;28(12):961–4. 10.3109/09513590.2012.683079 22621463

[10] Hadjadj L, Varbiro S, Horvath EM, Monori-Kiss A, Pal E, Karvaly GB, et al Insulin resistance in an animal model of polycystic ovary disease is aggravated by vitamin D deficiency: Vascular consequences. Diab Vasc Dis Res. 2018;15(4):294–301. 10.1177/1479164118758580 29465004

[11] Turetsky A, Goddeau RP Jr., Henninger N. Low Serum Vitamin D Is Independently Associated with Larger Lesion Volumes after Ischemic Stroke. J Stroke Cerebrovasc Dis. 2015;24(7):1555–63. 10.1016/j.jstrokecerebrovasdis.2015.03.051 26009498

[12] Thomson RL, Spedding S, Buckley JD. Vitamin D in the aetiology and management of polycystic ovary syndrome. Clin Endocrinol (Oxf). 2012;77(3):343–50. 22574874 10.1111/j.1365-2265.2012.04434.x

[13] Mahmoudi T. Genetic variation in the vitamin D receptor and polycystic ovary syndrome risk. Fertil Steril. 2009;92(4):1381–3. 10.1016/j.fertnstert.2009.05.002 19501823

[14] de Groot PC, Dekkers OM, Romijn JA, Dieben SW, Helmerhorst FM. PCOS, coronary heart disease, stroke and the influence of obesity: a systematic review and meta-analysis. Hum Reprod Update. 2011;17(4):495–500. 10.1093/humupd/dmr001 21335359

[15] Mozos I, Marginean O. Links between Vitamin D Deficiency and Cardiovascular Diseases. Biomed Res Int. 2015;2015:109275 10.1155/2015/109275 26000280 PMC4427096

[16] Balden R, Selvamani A, Sohrabji F. Vitamin D deficiency exacerbates experimental stroke injury and dysregulates ischemia-induced inflammation in adult rats. Endocrinology. 2012;153(5):2420–35. 10.1210/en.2011-1783 22408173 PMC3339639

[17] Labruijere S, van Houten EL, de Vries R, Musterd-Bagghoe UM, Garrelds IM, Kramer P, et al Analysis of the vascular responses in a murine model of polycystic ovary syndrome. J Endocrinol. 2013;218(2):205–13. 10.1530/JOE-13-0094 23734045

[18] Overpeck JG, Colson SH, Hohmann JR, Applestine MS, Reilly JF. Concentrations of circulating steroids in normal prepubertal and adult male and female humans, chimpanzees, rhesus monkeys, rats, mice, and hamsters: a literature survey. J Toxicol Environ Health. 1978;4(5–6):785–803. 10.1080/15287397809529700 104044

[19] Peinetti N, Scalerandi MV, Cuello Rubio MM, Leimgruber C, Nicola JP, Torres AI, et al The Response of Prostate Smooth Muscle Cells to Testosterone Is Determined by the Subcellular Distribution of the Androgen Receptor. Endocrinology. 2018;159(2):945–56. 10.1210/en.2017-00718 29194490

[20] Ding AQ, Stallone JN. Testosterone-induced relaxation of rat aorta is androgen structure specific and involves K+ channel activation. J Appl Physiol (1985). 2001;91(6):2742–50. 11717242 10.1152/jappl.2001.91.6.2742

[21] Perusquia M, Espinoza J, Montano LM, Stallone JN. Regional differences in the vasorelaxing effects of testosterone and its 5-reduced metabolites in the canine vasculature. Vascul Pharmacol. 2012;56(3–4):176–82. 10.1016/j.vph.2012.01.008 22326440 PMC3312741

[22] Quan A, Teoh H, Man RY. Acute exposure to a low level of testosterone impairs relaxation in porcine coronary arteries. Clin Exp Pharmacol Physiol. 1999;26(10):830–2. 10549414 10.1046/j.1440-1681.1999.03138.x

[23] Teoh H, Quan A, Leung SW, Man RY. Differential effects of 17beta-estradiol and testosterone on the contractile responses of porcine coronary arteries. Br J Pharmacol. 2000;129(7):1301–8. 10.1038/sj.bjp.0703164 10742284 PMC1571960

[24] Schmidt N, Brandsch C, Kuhne H, Thiele A, Hirche F, Stangl GI. Vitamin D receptor deficiency and low vitamin D diet stimulate aortic calcification and osteogenic key factor expression in mice. PLoS One. 2012;7(4):e35316 10.1371/journal.pone.0035316 22536373 PMC3335028

[25] Weng S, Sprague JE, Oh J, Riek AE, Chin K, Garcia M, et al Vitamin D deficiency induces high blood pressure and accelerates atherosclerosis in mice. PLoS One. 2013;8(1):e54625 10.1371/journal.pone.0054625 23349943 PMC3551761

[26] Dessapt-Baradez C, Reza M, Sivakumar G, Hernandez-Fuentes M, Markakis K, Gnudi L, et al Circulating vascular progenitor cells and central arterial stiffness in polycystic ovary syndrome. PLoS One. 2011;6(5):e20317 10.1371/journal.pone.0020317 21655296 PMC3105021

[27] Azziz R, Carmina E, Chen Z, Dunaif A, Laven JS, Legro RS, et al Polycystic ovary syndrome. Nat Rev Dis Primers. 2016;2:16057 10.1038/nrdp.2016.57 27510637

[28] Bajuk Studen K, Jensterle Sever M, Pfeifer M. Cardiovascular risk and subclinical cardiovascular disease in polycystic ovary syndrome. Front Horm Res. 2013;40:64–82. 10.1159/000341838 24002406

[29] Menezes AR, Lamb MC, Lavie CJ, DiNicolantonio JJ. Vitamin D and atherosclerosis. Curr Opin Cardiol. 2014;29(6):571–7. 10.1097/HCO.0000000000000108 25144342

[30] Masszi G, Horvath EM, Tarszabo R, Benko R, Novak A, Buday A, et al Reduced estradiol-induced vasodilation and poly-(ADP-ribose) polymerase (PARP) activity in the aortas of rats with experimental polycystic ovary syndrome (PCOS). PLoS One. 2013;8(3):e55589 10.1371/journal.pone.0055589 23555555 PMC3608629

[31] Lakhani K, Hardiman P, Seifalian AM. Intima-media thickness of elastic and muscular arteries of young women with polycystic ovaries. Atherosclerosis. 2004;175(2):353–9. 10.1016/j.atherosclerosis.2004.04.007 15262192

[32] Sara L, Nadasy G, Antal P, Szekeres M, Monori-Kiss A, Horvath EM, et al Arteriolar biomechanics in a rat polycystic ovary syndrome model—effects of parallel vitamin D3 treatment. Acta Physiol Hung. 2012;99(3):279–88. 10.1556/APhysiol.99.2012.3.5 22982716

[33] Varbiro S, Sara L, Antal P, Monori-Kiss A, Tokes AM, Monos E, et al Lower-limb veins are thicker and vascular reactivity is decreased in a rat PCOS model: concomitant vitamin D3 treatment partially prevents these changes. Am J Physiol Heart Circ Physiol. 2014;307(6):H848–57. 10.1152/ajpheart.01024.2013 25015958

[34] Hadjadj L, Pal E, Monori-Kiss A, Sziva RE, Korsos-Novak A, Maria Horvath E, et al Vitamin D deficiency and androgen excess result eutrophic remodeling and reduced myogenic adaptation in small cerebral arterioles in female rats. Gynecol Endocrinol. 2019:1–6. 10.1080/09513590.2018.1554037 30623742

[35] Luque-Ramirez M, Escobar-Morreale HF. Polycystic ovary syndrome as a paradigm for prehypertension, prediabetes, and preobesity. Curr Hypertens Rep. 2014;16(12):500 10.1007/s11906-014-0500-6 25304109

[36] Leman ES, Arlotti JA, Dhir R, Getzenberg RH. Vitamin D and androgen regulation of prostatic growth. J Cell Biochem. 2003;90(1):138–47. 10.1002/jcb.10605 12938163

[37] Bao BY, Hu YC, Ting HJ, Lee YF. Androgen signaling is required for the vitamin D-mediated growth inhibition in human prostate cancer cells. Oncogene. 2004;23(19):3350–60. 10.1038/sj.onc.1207461 15048085

[38] Murthy S, Agoulnik IU, Weigel NL. Androgen receptor signaling and vitamin D receptor action in prostate cancer cells. Prostate. 2005;64(4):362–72. 10.1002/pros.20251 15754350

[39] Krohn K, Haffner D, Hugel U, Himmele R, Klaus G, Mehls O, et al 1,25(OH)2D3 and dihydrotestosterone interact to regulate proliferation and differentiation of epiphyseal chondrocytes. Calcif Tissue Int. 2003;73(4):400–10. 10.1007/s00223-002-2160-9 12874696

[40] Somjen D, Kohen F, Amir-Zaltsman Y, Knoll E, Stern N. Vitamin D analogs modulate the action of gonadal steroids in human vascular cells in vitro. Am J Hypertens. 2000;13(4 Pt 1):396–403. 10821342 10.1016/s0895-7061(99)00203-4

[41] Ting HJ, Bao BY, Hsu CL, Lee YF. Androgen-receptor coregulators mediate the suppressive effect of androgen signals on vitamin D receptor activity. Endocrine. 2005;26(1):1–9. 10.1385/ENDO:26:1:001 15805579

[42] Wang WL, Tenniswood M. Vitamin D, intermediary metabolism and prostate cancer tumor progression. Front Physiol. 2014;5:183 10.3389/fphys.2014.00183 24860512 PMC4030193

[43] Ikeda Y, Aihara K, Yoshida S, Sato T, Yagi S, Iwase T, et al Androgen-androgen receptor system protects against angiotensin II-induced vascular remodeling. Endocrinology. 2009;150(6):2857–64. 10.1210/en.2008-1254 19196803

[44] Wilhelmson AS, Fagman JB, Johansson I, Zou ZV, Andersson AG, Svedlund Eriksson E, et al Increased Intimal Hyperplasia After Vascular Injury in Male Androgen Receptor-Deficient Mice. Endocrinology. 2016;157(10):3915–23. 10.1210/en.2016-1100 27533884

[45] Bourghardt J, Wilhelmson AS, Alexanderson C, De Gendt K, Verhoeven G, Krettek A, et al Androgen receptor-dependent and independent atheroprotection by testosterone in male mice. Endocrinology. 2010;151(11):5428–37. 10.1210/en.2010-0663 20861231

[46] Hanke H, Lenz C, Hess B, Spindler KD, Weidemann W. Effect of testosterone on plaque development and androgen receptor expression in the arterial vessel wall. Circulation. 2001;103(10):1382–5. 11245640 10.1161/01.cir.103.10.1382

[47] Li S, Li X, Li J, Deng X, Li Y, Cong Y. Experimental arterial thrombosis regulated by androgen and its receptor via modulation of platelet activation. Thromb Res. 2007;121(1):127–34. 10.1016/j.thromres.2007.03.008 17451792

[48] Masszi G, Novak A, Tarszabo R, Horvath EM, Buday A, Ruisanchez E, et al Effects of vitamin D3 derivative—calcitriol on pharmacological reactivity of aortic rings in a rodent PCOS model. Pharmacol Rep. 2013;65(2):476–83. 23744432 10.1016/s1734-1140(13)71023-5

[49] Geary GG, Krause DN, Duckles SP. Gonadal hormones affect diameter of male rat cerebral arteries through endothelium-dependent mechanisms. Am J Physiol Heart Circ Physiol. 2000;279(2):H610–8. 10.1152/ajpheart.2000.279.2.H610 10924060

[50] Kumral E, Bayulkem G, Evyapan D, Yunten N. Spectrum of anterior cerebral artery territory infarction: clinical and MRI findings. Eur J Neurol. 2002;9(6):615–24. 12453077 10.1046/j.1468-1331.2002.00452.x

[51] Sayeed I, Turan N, Stein DG, Wali B. Vitamin D deficiency increases blood-brain barrier dysfunction after ischemic stroke in male rats. Exp Neurol. 2019;312:63–71. 10.1016/j.expneurol.2018.11.005 30502340

